# Emerging therapies targeting the delta-like ligand 3 (DLL3) in small cell lung cancer

**DOI:** 10.1186/s13045-023-01464-y

**Published:** 2023-06-24

**Authors:** Charles M. Rudin, Martin Reck, Melissa L. Johnson, Fiona Blackhall, Christine L. Hann, James Chih-Hsin Yang, Julie M. Bailis, Gwyn Bebb, Amanda Goldrick, John Umejiego, Luis Paz-Ares

**Affiliations:** 1 Memorial Sloan Kettering Cancer Center, 1275 York Avenue, New York, NY 10065 USA; 2 Department of Thoracic Oncology, Airway Research Center North, German Center for Lung Research, LungenClinic Grosshansdorf, Grosshansdorf, Germany; 3 Department of Medical Oncology, Sarah Cannon Cancer Research Institute/Tennessee Oncology, PLLC, Nashville, TN USA; 4 Department of Oncology, The Christie NHS Foundation Trust, Manchester, UK; 5 Department of Oncology, Johns Hopkins University, Baltimore, MD USA; 6 Department of Oncology, National Taiwan University Hospital and National Taiwan University Cancer Center, Taipei, Taiwan; 7 Oncology Research, Amgen Inc., South San Francisco, CA USA; 8 Oncology TA-US, Amgen Inc., Thousand Oaks, CA USA; 9 Hospital Universitario 12 de Octubre, CNIO-H12o Lung Cancer Unit, Universidad Complutense and Ciberonc, Madrid, Spain

**Keywords:** DLL3, Small cell lung cancer, T-cell engager, Antibody-drug conjugate, Tarlatamab, AMG 757, Rovalpituzumab tesirine, BiTE

## Abstract

Small cell lung cancer (SCLC) is an aggressive neuroendocrine carcinoma with a poor prognosis. Initial responses to standard-of-care chemo-immunotherapy are, unfortunately, followed by rapid disease recurrence in most patients. Current treatment options are limited, with no therapies specifically approved as third-line or beyond. Delta-like ligand 3 (DLL3), a Notch inhibitory ligand, is an attractive therapeutic target because it is overexpressed on the surface of SCLC cells with minimal to no expression on normal cells. Several DLL3-targeted therapies are being developed for the treatment of SCLC and other neuroendocrine carcinomas, including antibody-drug conjugates (ADCs), T-cell engager (TCE) molecules, and chimeric antigen receptor (CAR) therapies. First, we discuss the clinical experience with rovalpituzumab tesirine (Rova-T), a DLL3-targeting ADC, the development of which was halted due to a lack of efficacy in phase 3 studies, with a view to understanding the lessons that can be garnered for the rapidly evolving therapeutic landscape in SCLC. We then review preclinical and clinical data for several DLL3-targeting agents that are currently in development, including the TCE molecules—tarlatamab (formerly known as AMG 757), BI 764532, and HPN328—and the CAR T-cell therapy AMG 119. We conclude with a discussion of the future challenges and opportunities for DLL3-targeting therapies, including the utility of DLL3 as a biomarker for patient selection and disease progression, and the potential of rational combinatorial approaches that can enhance efficacy.

**Supplementary Information:**

The online version contains supplementary material available at 10.1186/s13045-023-01464-y.

## Background

Small cell lung cancer (SCLC) is an aggressive, high-grade, neuroendocrine carcinoma (NEC) that annually contributes to 13%–15% of lung cancer diagnoses [1–3]. The prognosis for patients diagnosed with SCLC has been bleak; the 5-year survival rate ranges from 27% for those with localized disease to 3% for those with metastatic disease [1]. SCLC frequently presents at an advanced stage at the time of diagnosis and is characterized by rapid doubling time, a propensity to early metastasis, and transient responses to current standard-of-care (SOC) therapies that are almost always followed by the development of drug resistance and relapse [4, 5]. Cumulatively, these factors have led to SCLC being branded as a recalcitrant cancer, with the majority of patients failing to achieve long-term disease control with currently available therapies. To date, no targeted therapy for SCLC has proven to be better than existing therapies, even in trials with selected patient populations [6].

The current first-line SOC treatment for SCLC is platinum-based chemotherapy (cisplatin plus etoposide or carboplatin plus etoposide; CE) with concurrent radiotherapy for patients with limited-stage SCLC (LS-SCLC), followed by prophylactic cranial irradiation for patients who experience a complete response [7, 8], and CE with a programmed death-ligand 1 (PD-L1) inhibitor for patients with extensive-stage SCLC (ES-SCLC) [8]. In the US, topotecan (an antineoplastic, DNA-binding agent that induces lethal breaks in DNA) was the sole approved therapeutic agent for the second-line treatment of SCLC for more than two decades until lurbinectedin, an RNA polymerase inhibitor that inhibits active transcription, received accelerated approval for the treatment of relapsed SCLC in 2020 [7, 9, 10]. No therapeutic agent or regimen has received regulatory approval for the treatment of patients with SCLC who fail to respond or relapse after two or more lines of therapy. The limited improvement in outcomes with current therapies and the almost inevitable development of resistance and relapse following first-line chemotherapy serve to drive the ongoing search for more durably effective therapeutic approaches.

Immune checkpoint inhibitor (ICI) therapy has substantially improved outcomes for patients with non-small cell lung cancer and many other solid tumor types. Even though the high tumor mutation burden observed in SCLC has been correlated with an improved response to ICI therapy [2, 11], the addition of ICIs to first-line chemotherapy has provided transformative benefit in only a small subset of patients, with early retrospective analysis suggesting that the benefit may be confined to patients with inherently more inflamed tumors [12–14]. Several factors are thought to contribute to ICI resistance in SCLC, including downregulation of major histocompatibility complex (MHC) molecules, failure of antigen presentation, and high intratumoral heterogeneity [15, 16]. One strategy to bypass the lack of canonical antigen presentation pathways is to target an alternative cell surface protein on the cancer cell. Delta-like ligand 3 (DLL3) has emerged as an attractive tumor-specific target uniquely overexpressed on the cell surface of SCLC and other high-grade NECs [17]. Despite the apparent lack of benefit in short-term follow-up studies of ICIs in SCLC, it is important to note that emerging data from the long-term follow-up of patients with ES-SCLC from the CASPIAN and KEYNOTE-604 trials indicate that long-term maintenance treatment (> 3 years in the CASPIAN trial and up to 35 treatment cycles in the KEYNOTE-604 trial) with an ICI and chemotherapy was associated with significant improvement in survival (three times more patients alive at 3 years) compared with etoposide-platinum chemotherapy alone [12, 13].

In this review, we discuss various DLL3-targeting therapies starting with rovalpituzumab tesirine (Rova-T), the first DLL3-targeting antibody-drug conjugate (ADC), which advanced to phase 3 clinical studies before development was halted. We discuss the lessons that can be gleaned from the Rova-T clinical development program. We then summarize available preclinical and clinical data for the various DLL3-targeting therapeutic molecules currently in development and provide an overview of the different development stages of these programs with particular emphasis on the bispecific T-cell engager (TCE) tarlatamab (formerly AMG 757). We conclude with a perspective on the promise of DLL3-targeted therapeutics for the treatment of SCLC and other NECs.

## DLL3

**Fig. 1:**
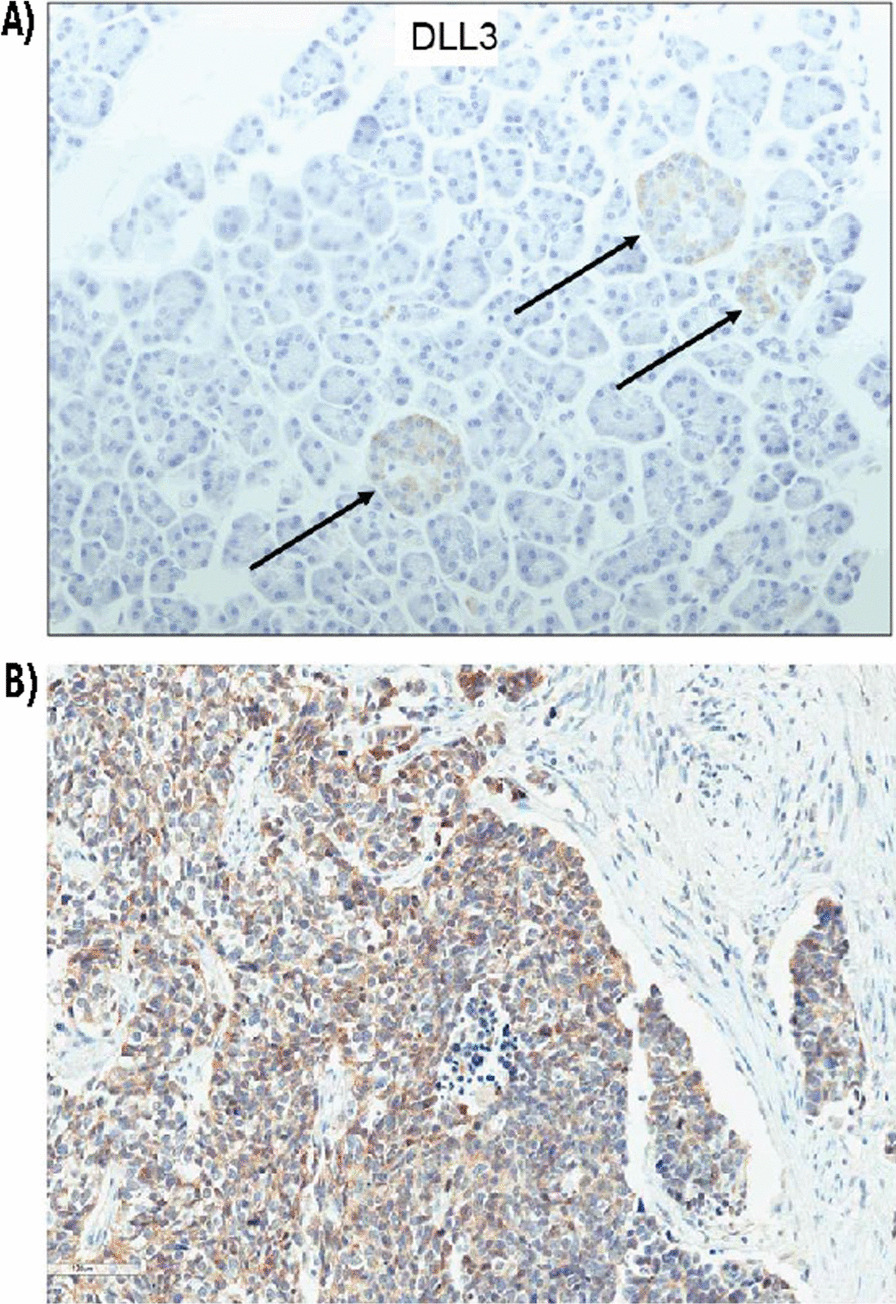
DLL3 expression in normal and tumor tissue. DLL3 protein expression via immunostaining (brown color) in **A** normal pancreatic tissue and **B** an SCLC tumor section [114]. Staining for DLL3 expression shows weak expression with a cytoplasmic pattern of localization in normal human pancreatic tissue sections (Panel **A**) and strong membranous and cytoplasmic expression in human SCLC (Panel **B**). Arrows in Panel A point to pancreatic islet cells. Blue hematoxylin counterstain is used to visualize cell nuclei. Original objective, ×200. *DLL3* delta-like ligand 3; *SCLC* small cell lung cancer

In the search for alternative therapeutic targets for SCLC, the transcription factor achaete-scute homolog 1 (ASCL1) has triggered particular interest due to its role as a key regulator of neuroendocrine differentiation and its ability to drive SCLC formation [18]. The increased expression of ASCL1 in some SCLC tumors (relative to the expression of the other neuroendocrine transcription factors NEUROD1 and POU2F3) has since led to the identification of a separate SCLC subtype, SCLC-A which is discussed in greater detail in a later section of the article. ASCL1 drives SCLC disease progression and cell survival by regulating the expression of several proto-oncogenes including *MYCL1, RET, SOX2, NFIB, and BCL2* [18, 19], as well as the *DLL3* gene, which encodes an inhibitory ligand that suppresses Notch signaling in SCLC cells [20]. The Notch pathway is an evolutionarily conserved pathway and Notch signaling in SCLC is implicated in multiple oncogenic cellular processes, such as cell proliferation, neuroendocrine cell plasticity, differentiation, chemoresistance, and modulation of the immune microenvironment [21]. DLL3 is an atypical Notch ligand whose overexpression promotes the growth of SCLC cells and enhances their migratory and invasive capacity [22]. DLL3 has also been implicated in establishing the metastatic- and treatment-resistant phenotype in NECs by promoting cell proliferation and the acquisition of resistance to platinum-doublet chemotherapy [23, 24]. DLL3 expression is low and mainly confined to the Golgi apparatus and cytoplasmic vesicles in normal cells but is upregulated and traffics to the surface of SCLC cells [25] (Fig. 1). Under physiological conditions, the transmembrane region and the flanking sequences in the DLL3 protein are thought to act as a retention signal confining the DLL3 protein to the Golgi membrane and cytoplasmic vesicles, with minimal-to-absent expression in normal cells [26]. Significant overexpression of the DLL3 protein leads to aberrant cell surface expression [26], as seen in SCLC, although the molecular mechanisms underlying DLL3 overexpression in transformed cells are not yet fully defined. Up to 85% of human SCLC tumors express the DLL3 protein on the cell surface [17, 27, 28]. The stability of DLL3 expression in SCLC tumors during therapy remains inconclusive. A study of 1073 SCLC tumors concluded that DLL3 expression was independent of sex, age, tumor stage, performance status, and number of prior lines of therapy [27]. In contrast, a much smaller study that examined DLL3 expression in 30 paired chemotherapy-naïve and chemotherapy-relapsed SCLC tumor samples found that DLL3 expression increased or decreased following chemotherapy in more than 40% of samples [28].

In addition to SCLC, DLL3 is also widely expressed in other NECs, such as pulmonary (certain molecular subtypes of large cell NEC [LCNEC]), gastroenteropancreatic, bladder, prostate, and cervical NECs [29]. High levels of DLL3 expression have been correlated with advanced disease and poor survival outcomes in these tumors [29].

The differential expression and localization profiles of DLL3 in normal and tumor cells render DLL3 an attractive, tumor-selective therapeutic target. Multiple approaches for targeting DLL3 are being explored preclinically and clinically (Table 1), including the bispecific TCE molecule tarlatamab, and other TCEs that have entered the clinical testing phase, such as HPN328, BI 764532, QLS31904, RO7616789, and PT217, as well as chimeric antigen receptor (CAR) constructs.

**Table 1:** Completed and ongoing clinical trials of DLL3-targeting therapies for SCLC^a^

Agent	Mechanism of action	Status^b^/trial identifier	Indications	Sponsor
*ADCs*				
Rovalpituzumab tesirine	ADC targeting DLL3	*Completed* NCT02674568 (phase 2) NCT01901653 (phase 1/2) NCT02874664 (phase 1) NCT03061812 (phase 3) NCT03086239 (phase 1)	Relapsed/refractory/recurrent/extensive-stage/advanced/metastatic SCLC/delta-like protein 3–expressing advanced solid tumors	AbbVie/Stemcentrx
		*Terminated or withdrawn* NCT02819999 (phase 1) NCT03026166 (phase 1/2) NCT03033511 (phase 3) NCT03334487 (phase 3) NCT02709889 (phase 1/2)		
SC-002	ADC targeting DLL3	*Terminated* NCT02500914 (phase 1)	Relapsed SCLC or large cell NEC	Stemcentrx
*CAR therapies*				
DLL3-CAR-NK cells	Anti-DLL3–transduced NK cells	*Recruiting* NCT05507593 (phase 1)	Relapsed/refractory extensive-stage SCLC	Tianjin Medical University Cancer Institute and Hospital
AMG 119	Anti-DLL3–transduced autologous T cells	*Suspended* NCT03392064 (phase 1)	Relapsed/refractory SCLC	Amgen Inc
*T-cell engagers*				
Tarlatamab	Half-life–extended DLL3 x CD3 bispecific T-cell engager	*Recruiting* NCT03319940 (phase 1; DeLLphi-300) NCT05060016 (phase 2; DeLLphi-301) *Active, not recruiting* NCT04885998 (phase 1; DeLLphi-302) *Not yet recruiting* NCT05740566 (phase 3; Dellphi-304)	Relapsed/refractory SCLC	Amgen Inc
		*Recruiting* NCT05361395 (phase 1; DeLLphi-303)	First-line treatment for extensive-stage SCLC	
BI 764532	DLL3/CD3 T-cell–engaging bispecific antibody	*Recruiting* NCT04429087 (phase 1)	Refractory, DLL3-expressing SCLC and other neuroendocrine neoplasms	Boehringer Ingelheim
HPN328	Tri-specific recombinant protein construct	*Recruiting* NCT04471727 (phase 1/2)	Relapsed/refractory, advanced DLL3-expressing malignancies	Harpoon Therapeutics
RO7616789	DLL3 x CD3/CD137 multispecific antibody	*Recruiting* NCT05619744 (phase 1)	Relapsed extensive-stage SCLC or high-grade NEC of any other origin	Hoffmann-La Roche
PT217	Anti-DLL3 x anti-CD47 bispecific antibody	*Not yet recruiting* NCT05652686 (phase 1)	Advanced or metastatic relapsed/refractory SCLC, large cell NEC, neuroendocrine prostate cancer, and gastroenteropancreatic neuroendocrine tumors	Phanes Therapeutics
QLS31904	Anti-DLL3 x anti-CD3 bispecific antibody	*Recruiting* NCT05461287 (phase 1)	Patients with advanced solid tumors, including SCLC, who progress on or are intolerant to standard of care, or have no effective standard therapeutic regimen	Qilu Pharmaceutical

^a^As registered on ClinicalTrials.gov

^b^Trial status as of March 2023

*ADC* antibody-drug conjugate, *CAR* chimeric antigen receptor, *CD* cluster of differentiation, *DLL3* delta-like ligand 3, *NEC* neuroendocrine carcinoma, *NK* natural killer, *SCLC* small cell lung cancer

## DLL3-targeting ADCs

### Mechanism of action

ADCs are typically composed of a humanized immunoglobulin G (IgG) molecule that is specific for a tumor-associated antigen (TAA) to which cytotoxic molecules (“warheads”) are attached by means of moieties called linkers. Linkers are generally designed to either cleave in special environments (e.g., low pH environment) or may require the presence of proteolytic enzymes, such as those found within a lysosome. ADCs bind to a cell surface–expressed TAA and are internalized via endocytosis [30]. Cleavage of the linker then allows for release of the cytotoxic warhead, which induces cellular apoptosis by either damaging DNA or inhibiting microtubule assembly.

### Rova-T

**Fig. 2:**
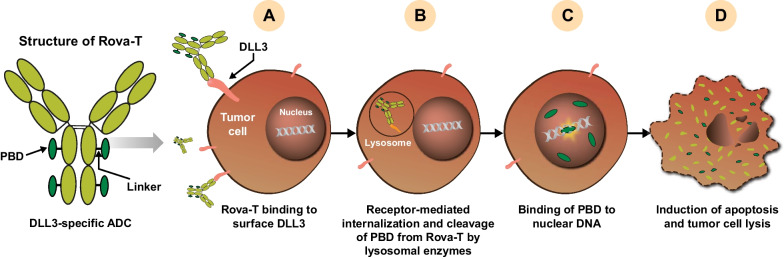
Mechanism of action of Rova-T. **A** Binding of Rova-T to cell surface DLL3 triggers receptor-mediated endocytosis and **B** internalization of the Rova-T–DLL3 complex followed by fusion with the late endosome. **C** PBD is released from the Rova-T complex following enzymatic cleavage within the lysosome. The released PBD intercalates between double-stranded nuclear DNA in a site-specific manner and causes DNA damage, which ultimately leads to **D** apoptosis [17]. *ADC* antibody-drug conjugate, *DLL3* delta-like ligand 3, *PBD* pyrrolobenzodiazepine, *Rova-T* rovalpituzumab tesirine

Rova-T is an ADC comprising a DLL3-specific humanized monoclonal antibody (SC16) conjugated to a membrane-permeable pyrrolobenzodiazepine (PBD) dimer toxin (warhead) via a lysosomal, protease-sensitive dipeptide linker [17, 31]. Binding of Rova-T to cell surface DLL3 causes internalization of the ADC-target complex by endocytosis. Rova-T’s valine-alanine linker is subsequently cleaved by lysosome-associated cathepsin B, releasing PBD into the cytoplasm. PBD then enters the nucleus, cross-links DNA, and induces tumor cell death by apoptosis [32] (Fig. 2).

#### Preclinical experience

In preclinical studies, intraperitoneal administration of Rova-T inhibited tumor progression in patient-derived xenograft (PDX) models of SCLC with a time to tumor progression (TTP) of 132 days in comparison with cisplatin treatment, which had a TTP of only 4 days [17]. Rova-T also showed activity against PDX tumor models that were refractory to CE treatment, suggesting potential utility in the context of platinum-refractory SCLC [17].

In preclinical toxicology studies, nonhuman primates (NHPs) treated with high and medium doses of Rova-T developed skin thickening, hyperpigmentation, mild kidney degeneration, and reversible myelosuppression [17]. The toxicology profiles observed in NHPs predicted some of the clinical adverse events (AEs) as described below.

#### Clinical experience

Rova-T has been administered to more than 1000 patients as monotherapy and as combination therapy with other chemotherapy and immunotherapy treatments in at least 10 clinical trials, including two phase 3 clinical trials. The first-in-human (FIH) study of Rova-T yielded an objective response rate (ORR) of 18%, which increased to 38% in DLL3-high patients with SCLC (patients whose tumors expressed DLL3 on ≥ 50% of cells by immunohistochemistry [IHC]) [31]. This impressive preliminary response rate appeared to be similar irrespective of whether the patient was treated in the second- or third-line setting. The observed efficacy led to the initiation of subsequent phase 2 and phase 3 trials of Rova-T.

##### Safety profile

In phase 1–3 trials of Rova-T in patients with recurrent SCLC after platinum-based chemotherapy, grade ≥ 3 treatment-emergent AEs (TEAEs) were observed in 38%–64% of patients with SCLC, and grade 5 events were observed in 1.7%–7.1% of patients [6, 24, 31, 33]. Serious TEAEs were reported in 30%–56% of patients. Thrombocytopenia, pleural effusion, photosensitivity reactions, and anemia were among the most frequently encountered TEAEs with Rova-T.

Treatment discontinuations as a result of AEs were reported in 10%–22% of Rova-T–treated patients in clinical studies [6, 31, 33–35]. Pleural and pericardial effusions, thrombocytopenia, and maculopapular rash were the most frequent causes for treatment discontinuation [31, 33]. Rova-T–related AEs were typically managed by dose reductions, dose interruptions, treatment discontinuations, and other symptom-specific, risk-management protocols [31, 33, 35]. The lack of a definitive understanding of the pathophysiological mechanisms underlying Rova-T toxicity and the high incidence of AEs may have contributed to the high discontinuation rates observed with this ADC.

The toxicity profile seen with Rova-T can be predominantly attributed to the PBD warhead, as clinical studies of other ADCs linked to PBD have reported a similar toxicity profile (hematological abnormalities, skin toxicities, and pericardial/pleural effusions) [36]. Two potential mechanisms, which are not mutually exclusive, may contribute to the exposure of nonmalignant tissues to the cytotoxic moiety of ADCs. First, premature cleavage of the ADC linker by tumor cells, tumor-associated macrophages, or other sources of cathepsin B may result in systemic toxicity through the nonspecific release of the warhead before it can be internalized by the target cell [6, 33]. Second, a “bystander” effect, in which diffusion of the cytotoxic warhead from disintegrating tumor cells and its subsequent uptake by healthy, nontarget cells resulting in the nonspecific lysis of cells, may potentially contribute to ADC-associated toxicity [33, 37]. The bystander effect can be a double-edged sword as it not only amplifies the therapeutic effect of ADCs by killing neighboring target-negative tumor cells (thereby partially mitigating the challenges of tumor heterogeneity) but can also cause the death of healthy, nonmalignant cells, leading to systemic AEs.

##### Efficacy profile

In the FIH study of Rova-T–treated patients with recurrent/progressive SCLC, previously treated with one or two chemotherapy regimens, including a platinum-based regimen, the 1-year survival rate was 36% compared with the 14% survival rate observed in historical studies of conventional chemotherapy in the third-line setting [31, 38]. The promising ORR and 1-year survival rates coupled with the lack of effective treatments in the third-line setting warranted further clinical evaluation of Rova-T.

In the phase 2 TRINITY study investigating Rova-T as third-line and beyond therapy in 339 patients with DLL3-expressing SCLC (≥ 1% of DLL3-expressing tumor cells), the ORR was 12.4% and the median overall survival (OS) was 5.6 months [33]. In patients with high DLL3 expression (DLL3 expressed by > 75% of tumor cells by IHC), the ORR was slightly higher at 14.3%, with a median progression-free survival (PFS) of 3.8 months and a median OS of 5.7 months.

The TAHOE (Rova-T vs topotecan as second-line therapy) and MERU (Rova-T as maintenance therapy after first-line therapy vs placebo) phase 3 studies were terminated early as they did not meet the prespecified interim primary PFS and/or OS endpoints [6, 35]. In the TAHOE study, the median OS was 6.3 months in the Rova-T arm and 8.6 months in the topotecan arm [6]. In the MERU study, the median OS was 8.8 months in the Rova-T arm and 9.9 months in the placebo arm; limiting the analysis to the subset of DLL3-high (≥ 75% DLL3-positive tumor cells) patients did not improve the efficacy [35]. These clinical findings and other strategic considerations led to the discontinuation of Rova-T development [39].

A summary of available clinical and preclinical data from DLL3-targeting development programs is presented in Table 2.

**Table 2:** Overview of DLL3-targeting development programs

	Preclinical data	SCLC clinical program status^a^	Clinical safety	Clinical efficacy
*DLL3-targeting T-cell engagers*				
Tarlatamab	In PDX studies, tarlatamab caused significant tumor regression (83%–98%) and a significant reduction in tumor volume [59] In a disseminating orthotopic model of SCLC, tarlatamab-induced significant tumor growth inhibition at a low mg/kg weekly dose [59] In exploratory toxicology studies in NHPs, tarlatamab induced a transient increase in heart rate, a transient minor decrease in lymphocyte frequency, and a mild infiltration of lymphocytes and eosinophils into the pituitary [60]	Administered to > 100 patients in an ongoing FIH phase 1 study as second-line (and beyond) treatment for SCLC Phase 1 combination studies with anti–PD-1 and anti–PD-L1 (with or without platinum-etoposide) in ES-SCLC are ongoing Phase 2 study in SCLC is ongoing Phase 3 study comparing tarlatamab with SOC chemotherapy for patients with relapsed SCLC will begin patient recruitment shortly	Phase 1 (NCT03319940) results: TRAEs in 90.7%; grade ≥ 3 in 30.8% CRS (52.3%), pyrexia (37.4%), dysgeusia (22.4%), fatigue (21.5%), and nausea (19.6%) were the most commonly observed TRAEs Most CRS events occurred in the first treatment cycle and were managed with supportive care, corticosteroids, and tocilizumab when necessary Other adverse events of special interest (based on Amgen’s MedDRA query narrow safety reporting definitions) included neurological events and neutropenia Treatment-related neurologic events, 49.5% (grade ≥ 3, 6.5%); treatment-related neutropenia, 15.9% (grade ≥ 3, 9.3%)	Results from the phase 1 study: Confirmed ORR of 23.4% (including two [1.9%] complete responses and 23 [21.5%] partial responses) Disease control rate of 51.4% Median duration of response of 12.3 months Median PFS of 3.7 months and median OS of 13.2 months
HPN328	HPN328-mediated T-cell–dependent cellular cytotoxicity against the DLL3-positive, NCI-H82 cell lines and against DLL3-expressing cynomolgus cells [57, 62] HPN328 inhibited the growth of NCI-H82 xenografts HPN328 was well-tolerated in NHPs at 1 mg/kg and 10 mg/kg and had a half-life of 2.8–3.3 days [61, 62]	Administered to 18 patients so far in a phase 1 study [72] Maximum tolerated dose not yet reached; dose escalation ongoing	Dysgeusia, fatigue, and hypotension seen in seven patients (39%) each [72] CRS transient and manageable, with 22% of patients experiencing grade 1–2 CRS; no grade ≥ 3 CRS reported [72]	Three of eleven (27%) patients with SCLC had > 30% decreases in sum of target lesion diameters, including one confirmed partial response [72] Four of six (67%) patients treated at ≥ 1.215 mg/week had a decrease in sum of target lesion diameters [72]
BI 764532	BI 764532 induced T-cell lysis of several DLL3-expressing SCLC cell lines in a dose-dependent manner [55] BI 764532 induced T-cell infiltration into tumors, tumor regression, and tumor growth inhibition in a CD3 + T-cell humanized mouse model with an SHP-77 xenograft [55]	Phase 1, dose-escalation study in patients with SCLC, large cell neuroendocrine carcinoma, neuroendocrine carcinoma, or small cell carcinomas is ongoing [110]	Results not yet published	Results not yet published
QLS31904	QLS31904 inhibited tumor growth in triple-immunodeficient mice implanted with SHP-77 and human T cells [112] Well-tolerated in NHPs at five weekly doses of 10 mg/kg [112]	Patient recruitment for a phase 1 study ongoing	Unknown	Unknown
*Antibody-drug conjugates*				
Rovalpituzumab tesirine (Rova-T)	In PDX models, mice treated with Rova-T demonstrated complete and durable responses against chemotherapy-resistant and recurrent tumors in contrast to mice treated with cisplatin and etoposide [17] In NHPs, Rova-T treatment was associated with reversible myelosuppression, mild kidney degeneration, and skin thickening and hyperpigmentation [17]	Administered to > 1000 patients across more than 10 studies, including two phase 3 studies Clinical development discontinued due to a lack of survival benefit in phase 3 studies	Results across multiple studies: Thrombocytopenia, pleural effusions, photosensitivity reactions, and anemia were the most frequently encountered TRAEs Toxicity attributed to the cytotoxic warhead—PBD Adverse events managed by dose reductions, treatment interruptions, treatment discontinuations, and symptom-specific management	Response rates of 12%–18% in the initial phase 1 and 2 studies [31, 33] Randomized phase 3 studies failed to show a benefit with Rova-T: Phase 3 TAHOE: Median OS: Rova-T (6.3 months) vs topotecan (8.6 months); ORR: Rova-T (15%) vs topotecan (21%) [6] Phase 3 MERU: OS: Rova-T (8.8 months) vs topotecan (9.9 months); ORR: Rova-T (9%) vs topotecan (4%) [35]
SC-002	Unknown/not published	Administered to 35 patients with relapsed/refractory SCLC in an FIH study [113] Study terminated with no further development planned	Commonly occurring TEAEs were dyspnea (43%), pleural effusions (43%), and decreased appetite (34%) [113]	Five patients (14%) achieved a partial response [113] No patient achieved a complete response Fourteen (40%) patients experienced stable disease Eleven patients had progressive disease
*CAR therapies*				
AMG 119	Exhibited cytotoxic activity against DLL3-expressing cells and SCLC cell lines at low effector-target ratios [73] Induced complete tumor regression in female NOD SCID mice with SHP-77 xenografts [73]	Five patients treated in an FIH study [74]	Reported TRAEs included pneumonitis, seizure, supraventricular tachycardia, and anemia [74]	One of five patients (20%) achieved a confirmed partial response, two patients (40%) achieved stable disease, one patient had progressive disease, and one patient’s response was unevaluable [74]
DLL3-CAR- NK-92 cells	DLL3-CAR-NK-92 cells induced tumor regression in a metastatic SCLC model and a subcutaneous tumor model [76] DLL3-CAR-NK-92 cell infiltration observed in subcutaneous tumor sites [76]	Recruiting patients with relapsed/refractory ES-SCLC for a phase 1 trial	Unknown	Unknown

^a^As updated on ClinicalTrials.gov

*CAR* chimeric antigen receptor, *CD* cluster of differentiation, *CRS* cytokine release syndrome, *DLL3* delta-like ligand 3, *ES-SCLC* extensive-stage SCLC, *FIH* first-in-human, *MedDRA* Medical Dictionary for Regulatory Activities, *NHP* nonhuman primate, *NK* natural killer, *NOD SCID* nonobese diabetic/severe combined immunodeficiency, *ORR* objective response rate, *OS* overall survival, *PBD* pyrrolobenzodiazepine, *PD-1* programmed cell death protein-1, *PD-L1* programmed death-ligand 1, *PDX* patient-derived xenograft, *PFS* progression-free survival, *Rova-T* rovalpituzumab tesirine, *SCLC* small cell lung cancer, *SOC* standard-of-care, *TEAE* treatment-emergent adverse event, *TRAE* treatment-related adverse event

## TCEs

Despite more than two decades of clinical testing, only four TCEs have received the US Food and Drug Administration (FDA) approval to date. Blinatumomab, a TCE that targets the cluster of differentiation (CD)19 antigen, was approved for the treatment of Philadelphia chromosome (Ph)-negative relapsed/refractory B-cell precursor acute lymphoblastic leukemia (B-ALL) in 2014 and for the treatment of Ph-positive relapsed/refractory B-ALL in 2017 [40]. In 2022, three TCE molecules were approved: tebentafusp-tebn for the treatment of unresectable or metastatic uveal melanoma [41], mosunetuzumab-axgb for the treatment of relapsed/refractory follicular lymphoma [42], and teclistamab-cqyv for relapsed/refractory multiple myeloma [43]. TCEs continue to advance in clinical development across multiple tumor indications and are expected to become an important component of anticancer strategies. Of the TCEs that are currently in development for SCLC, tarlatamab is the most advanced, having entered phase 3 in 2023.

### Mechanism of action

**Fig. 3:**
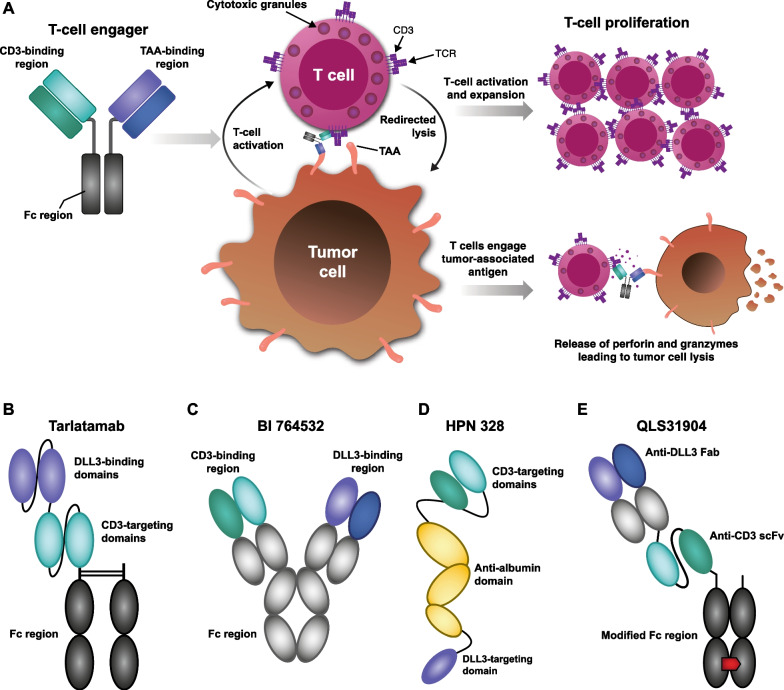
Mechanism of action of **A** TCEs and **B**–**E** structure of DLL3-targeting TCEs in development. Panel **A** depicts a generic structure for T-cell engagers, although it should be noted that Fc regions are not a feature of all DLL3-targeting T-cell engagers. The structures of the newer DLL3-targeting T-cell engagers RO7616789 and PT217 have not yet been published. *CD* cluster of differentiation; *DLL3* delta-like ligand 3, *Fab* fragment antigen-binding, *Fc* fragment crystallizable, *scFv* single-chain variable fragment, *TAA* tumor-associated antigen, *TCE* T-cell engager, *TCR* T-cell receptor

TCEs have dual specificities, a characteristic that allows them to simultaneously bind to the CD3 complex on T cells and a target antigen on tumors [44]. This dual binding brings tumor cells into close proximity with autologous T cells, triggers the formation of an immunological synapse and T-cell activation, and initiates a polyclonal T-cell response that is characterized by CD3 clustering, T-cell proliferation, and the release of pore-forming granzyme and perforin [45–47]. This sequence of events can culminate in tumor cell apoptosis and amplification of the T-cell response (Fig. 3A).

A characteristic feature of TCE molecules is MHC-I–independent T-cell activation, which may be an advantage in solid tumors that evade effective immune surveillance by downregulating surface expression of MHC-I [48]. Other features of TCEs that can prove advantageous include the ability to employ the entire T-cell repertoire against any cell that expresses the relevant target and a capacity to induce T-cell–mediated killing at very low concentrations [49–51].

### DLL3-targeting TCEs

#### Tarlatamab

Tarlatamab is a bispecific TCE with dual affinity for DLL3 on tumor cells and CD3 on T cells. The tarlatamab molecule consists of two single-chain variable fragments (scFv) connected by a short, flexible linker and includes a stable, effector-functionless fragment crystallizable (Fc) domain to increase the serum half-life (Fig. 3B). Tarlatamab is designed to form a cytolytic synapse by simultaneously binding to tumor cells and T cells, which is associated with T-cell activation, transient cytokine production, and T-cell proliferation. Activated T cells release pore-forming enzymes, such as perforin and granzyme B, which cause tumor cell apoptosis [52, 53]. The activated T cells also proliferate, increasing the number of effector T cells in the vicinity of the tumor and amplifying the antitumor effect [54].

#### Other TCEs

Other DLL3/CD3 TCEs have similar mechanisms of action to tarlatamab, namely, the redirection of T cells to kill DLL3-expressing tumor cells. BI 764532 is a bispecific DLL3/CD3 antibody that has an IgG-like scaffold (Fig. 3C) [55]. HPN328 is a Tri-specific T Cell-Activating Construct (TriTAC) comprising three humanized antibody-derived binding domains: an N-terminal domain that binds DLL3 on tumor cells, a middle domain that binds to human serum albumin (for half-life extension), and a C-terminal domain that binds to CD3 (Fig. 3D) [56]. TriTACs differ from other TCEs in having a much smaller molecular weight due to the use of single-domain antibodies for binding to the TAA and human serum albumin [56, 57]. QLS31904 is another DLL3/CD3 bispecific TCE for which clinical evaluation is planned. This TCE comprises three chains covalently linked by disulfide bonds: an anti–DLL3-specific fragment antigen-binding (Fab) component, an anti–CD3-directed scFv fragment, and a modified Fc region to support heterodimerization and purification and prevent unwanted Fc receptor binding (Fig. 3E) [58].

### Preclinical experience

#### Tarlatamab

In preclinical studies, tarlatamab monotherapy promoted significant tumor regression and complete antitumor responses in biologically relevant models of primary and metastatic SCLC [59]. Tarlatamab exhibited antitumor activity in orthotopic SHP-77 tumors and cleared liver metastases in the NCI-H82 model of metastatic SCLC. Tarlatamab treatment led to 83% and 98% tumor regressions in two different PDX models of SCLC engrafted with human T cells. Consistent with its expected mechanism of action, tarlatamab promoted CD4 + and CD8 + T-cell infiltration into PDX SCLC tumors, T-cell activation, production of inflammatory cytokines, and the release of cytotoxic granules in vitro [59]. Tarlatamab-induced granzyme B production and cytotoxicity occurred concurrently with the release of cytokines such as interferon (IFN)-γ, interleukin (IL)-6, IL-10, tumor necrosis factor (TNF)-α, and IL-4 [59].

Tarlatamab was well-tolerated in NHPs with no treatment-related AEs (TRAEs) up to the highest dose level tested (4.5 mg/kg administered weekly). Tarlatamab induced transient decreases in lymphocyte populations at a high dose (4.5 mg/kg), and the immune cell infiltration into the pituitary (an organ that expresses low levels of DLL3) was consistent with target engagement. The good tolerability in healthy NHPs underscores the low, mainly cytoplasmic expression of DLL3 in normal tissue [59, 60]. Tarlatamab has a mean half-life of 234 h (9.8 days) in NHPs, supporting intermittent dosing in the clinical setting [59].

#### BI 764532

As observed with tarlatamab, BI 764532 demonstrated DLL3-dependent antitumor activity in preclinical models of SCLC. BI 764532 induced the specific lysis of DLL3-expressing SHP-77 cells at effector-target ratios ranging from 2:1 to 30:1 with maximal activity at the ≥ 10:1 ratio [55]. BI 764532 could redirect CD4 + and CD8 + T cells to lyse DLL3-expressing cells with more potent cytotoxic activity observed in cells with higher DLL3 expression levels. In vivo, BI 764532 induced significant tumor growth inhibition and sustained tumor regression when compared with the vehicle-only control in CD3 + T-cell humanized mice bearing subcutaneous SHP-77 xenograft tumors. Analysis of tumor tissue from mice treated with BI 764532 revealed infiltration of CD3 + T cells into tumor tissue, including increased numbers of both CD4 + and CD8 + T cells within tumors compared with the vehicle-only controls. BI 764532 demonstrated similar pharmacokinetics to tarlatamab in NHPs, with a half-life of 10 days [55].

#### HPN328

HPN328 also demonstrated dose-dependent, DLL3-specific, T-cell–dependent, cellular cytotoxicity against DLL3-expressing SCLC cells. HPN328 induced the dose-dependent upregulation of CD25 and CD69 on T cells and the secretion of TNF-α and IFN-γ in the presence of DLL3-expressing tumor cells, consistent with the expected mechanism of action [61]. HPN328 mediated significant growth inhibition of subcutaneous NCI-H82 SCLC xenograft tumors in mice. HPN328 was well-tolerated in NHPs at doses of 1 mg/kg and 10 mg/kg, with no adverse biochemical changes or clinically significant changes on necropsy. In NHPs, HPN328 exhibited a serum half-life between 2.7 and 3.5 days [61, 62].

### Clinical experience

#### Tarlatamab

Tarlatamab is being evaluated in six ongoing clinical studies, including five trials in patients with SCLC (phases 1–3; Table 1) and a phase 1 trial in patients with neuroendocrine prostate cancer (NEPC).

##### Safety profile

In the ongoing DeLLphi-300 FIH trial of tarlatamab (NCT03319940), 90.7% (97/107) of patients experienced TRAEs of any grade and 30.8% (33/107) experienced grade ≥ 3 TRAEs [63]. Four patients (3.7%) discontinued tarlatamab due to TRAEs (pneumonitis [n = 2], immune effector cell–associated neurotoxicity syndrome, and encephalopathy [n = 1 each]). No grade 5 TRAEs were identified. (Further investigation into a previously reported grade 5 TRAE due to pneumonitis led to a change in the etiology and causality of this event, which has now been deemed to be a grade 5 TEAE lung infection.) Cytokine release syndrome (CRS) was the most common TRAE and was seen in 52.3% (56/107) of patients. Of these, 73.2% (41/56) of patients experienced grade 1 CRS, and only one patient developed grade 3 CRS. Grade > 3 CRS events were not observed. Tarlatamab-associated CRS was characterized by transient mild fever and/or hypotension that generally did not require vasopressor support [64] and typically occurred in the first treatment cycle; < 4% of patients experienced recurrent CRS in the second cycle or later. Tarlatamab-associated CRS was manageable with supportive care, including acetaminophen or paracetamol, intravenous fluids, supplemental oxygen (where required), and in some cases, tocilizumab (anti–IL-6 monoclonal antibody); however, only 8/107 patients (7.5%) required the use of tocilizumab for CRS in this trial at the time of data cutoff.

In addition to CRS, neurological events also emerged as events of interest. Treatment-related neurologic AEs occurred in 53 patients (49.5%) and were mostly grade 1 in severity, with dysgeusia (22.4%), headache (10.3%), confusional state (5.6%), and dizziness (5.6%) being the most commonly reported AEs. Treatment-emergent grade ≥ 3 neurological events occurred in 12 patients (11.2%), including confusional state (n = 5) and encephalopathy (n = 2), all of which resolved. Grade 4 confusion (n = 1) was the only grade > 3 neurological event that was observed. Neutropenia, an AE found to be associated with tarlatamab, was unexpected based on preclinical data; the mechanism is not currently understood. Treatment-related neutropenia was observed in 15.9% (17/107) of patients; grade ≥ 3 neutropenia was observed in 9.3% (10/107) of patients. Febrile neutropenia occurred in one patient and was not considered to be treatment related.

CRS was expected with tarlatamab, given its mechanism of action and based on clinical experience with other TCE molecules. While the molecular mechanisms of CRS are not completely understood, it can develop from activation of endothelial cells and bystander immune cells after TCE binding [65]. Activated T cells, monocytes, and macrophages produce supraphysiological quantities of IFN-γ, IL-6, and TNF-α, which collectively trigger an inflammatory cycle that can overpower the homeostatic mechanisms in the host [65]. It is not clear which, if any, of the cytokines induced by TCEs are required for their antitumor activity in patients [66].

The mechanism of neurological toxicity in the tarlatamab (FIH) study is not fully understood, as toxicology studies in NHPs did not reveal tarlatamab-related neurological signs or histopathological evidence of neurotoxicity [59, 60]. Neurological AEs have been observed with the TCEs blinatumomab, mosunetuzumab, and teclistamab [67–69], but the extent to which expression of the targeted tumor antigen in neural tissue informs the potential neurotoxicity of TCEs, relative to other factors, needs to be explored further. In other studies, TCE-associated neurotoxicity has been linked to T-cell–mediated inflammatory events in the perivascular space within the brain [70, 71]. Strategies such as step-dosing, premedication with corticosteroids, and fluid administration have been employed in ongoing clinical trials for mitigating CRS and neurological events and will be critical for successful clinical adoption. The neutropenia observed with tarlatamab treatment warrants further study, especially if tarlatamab is used in combination with bone marrow–suppressing agents.

Tarlatamab is administered in an inpatient setting as the current clinical trial protocols for tarlatamab require 48-h monitoring on days 1 and 8 of cycle 1, but the encouraging FIH safety profile has led to the ongoing exploration of reduced monitoring in phase 2 (20–24 h and 24 h monitoring) and phase 1 (6–8 h and 8 h monitoring) settings.

##### Efficacy profile

At the latest data cutoff on July 19, 2022, the FIH study of tarlatamab had enrolled 107 patients with progressive or recurrent SCLC following at least one prior line of therapy (2L +), including platinum-based chemotherapy, across 10 dose levels ranging from 0.003 mg to 100 mg [63]. Step-dosing was adopted starting with the 3-mg cohort (using 1 mg as the run-in dose followed by the target dose on day 8, day 15, and once every 2 weeks thereafter) to mitigate the CRS observed in prior cohorts. Responses were seen starting at the 0.3-mg dose level, with a greater number of responses in the ≥ 3-mg dose levels. Confirmed responses were seen in 25 patients (ORR: 23.4%), which included two patients (1.9%) with complete responses and 23 patients (21.5%) with partial responses (PR). Stable disease (SD) was seen in 30 patients (28%). The disease control rate was 51.4%, median PFS was 3.7 months (95% confidence interval [CI]: 2.1–5.4), and the median OS was 13.2 months (95% CI: 10.5–not estimable). Among confirmed responders, the median time to response was 1.8 months (range: 1.2–7.4 months) and the median duration of response (DOR) was 12.3 months (95% CI: 6.6–14.9), indicating that in most responders, a response could be observed as early as by their first scan with a response duration that was encouraging relative to that observed in other trials of SCLC therapies.

### HPN328

Preliminary results from the phase 1 trial of HPN328 have been presented [72]. As of April 21, 2022, 18 patients with SCLC and other NECs had been treated with doses ranging from 0.015 mg/week to 12.0 mg/week with step-dosing utilized at higher doses. In total, 3 of 11 patients (27%) with SCLC experienced a > 30% decrease in the sum of target lesion diameters, including one patient with SCLC who experienced a confirmed PR. Overall, 33% had SD [72]. Grade 1–2 CRS was seen in 22% of patients; grade > 3 CRS events were not observed at the tested dose levels, although dose optimization is ongoing and the maximum tolerated dose has not been reached. Treatment duration ranged from 4.1 to 41.4 weeks.

Other DLL3-targeting TCEs and molecules, including BI 764532, QLS31904, RO7616789, and PT217, have entered phase 1 clinical trials and are being evaluated in patients with DLL3-positive SCLC and other neuroendocrine tumors (NETs; Table 1, Table 2). Clinical data for these TCEs are not yet available.

## DLL3-targeting CAR therapies

The success of CAR T-cell therapies in the treatment of hematological malignancies has prompted considerable interest in evaluating their efficacy in solid tumors. The identification of DLL3 as a tractable tumor-specific target has led to the design and testing of DLL3-targeting CAR therapies in SCLC.

### AMG 119

AMG 119 is a genetically engineered T cell that is generated by transducing autologous T cells with a self-inactivating lentiviral vector that encodes an anti-DLL3 target-binding domain, a CD28 and 4-1BB co-stimulatory domains, and a CD3 domain. In preclinical studies, AMG 119 exhibited specific cytotoxic activity against DLL3-expressing SCLC cells and antitumor activity in SCLC xenograft models [73, 74].

A phase 1 clinical trial of AMG 119 in five patients with relapsed/refractory SCLC revealed no dose-limiting toxicities or grade ≥ 4 AEs [74]. One patient experienced a confirmed PR with 43% reduction in the sum of target lesion diameters from baseline, while another experienced 16% reduction in the sum of target lesion diameters and disappearance of multiple liver metastases but did not qualify as a responder per the Response Evaluation Criteria in Solid Tumors (RECIST) v1.1 criteria. AMG 119 CAR T cells were detectable for up to 86 days after infusion. These preliminary data provide proof-of-principle for the further development of DLL3-targeting CAR T-cell therapies in SCLC.

Other DLL3-targeting CAR T-cell therapies for SCLC, such as LB2102 and ALLO-213, are in development but are yet to initiate clinical testing.

### DLL3-targeting CAR-NK therapies

CAR-transduced natural killer (NK)-92 cells have demonstrated potent and specific lytic activity in preclinical studies and have additional advantages, such as a donor-independent manufacturing process and “off-the-shelf” availability [75]. NK-92 cells that were transduced with a vector encoding the anti-DLL3 scFv domain, an NKG2D transmembrane domain, and a 2B4-CD3 domain exhibited specific antitumor activity against DLL3-positive cell lines and induced tumor regression in a pulmonary metastasis tumor model in immunodeficient mice [76]. A phase 1 trial of DLL3-CAR-NK cells in patients with relapsed/refractory ES-SCLC has recently begun patient recruitment (NCT05507593).

## Clinical outlook

### DLL3-targeting TCEs

TCEs have certain distinct advantages, especially in the context of SCLC biology. SCLC demonstrates multiple characteristics that may promote escape from the host immune response, including downregulation of MHC-I expression, establishment of an immunosuppressive tumor microenvironment, and negative regulation of cytotoxic T cells [15]. Importantly, TCEs activate T cells independent of MHC class I molecules and PD-L1 expression. Other advantages of TCEs include (1) the requirement for co-engagement of a target cell with the effector cell for activity, thereby preventing nonspecific activation of effector cells, (2) the lack of requirement for prior stimulation of T cells or in vivo co-stimulation, (3) the small size of many TCEs, which can bring together target and effector cells in close proximity, thereby enabling efficient lysis of tumor cells, and (4) off-the-shelf availability [77].

In preclinical studies, anti-DLL3 TCEs have shown strong binding ability to and potent lytic ability even against cells that expressed low levels of surface DLL3 (< 1000 molecules/cell) [59]. This suggests that these agents may exert an antitumor effect even in the case of low levels of DLL3 expression in some tumor cells—if a sufficient number of T cells are present.

The safety profiles and the management protocols for AEs observed with TCEs and ADCs are markedly different. In general, TCE-associated toxicities derive from their immunostimulatory mechanism of action, while ADC-associated toxicities are related to the cytotoxic warhead. Tarlatamab-related AEs typically resolved either with dose modification, a temporary cessation of treatment, corticosteroid use, administration of anti–IL-6 therapy, or spontaneously and have been thus far manageable from a clinical perspective. The availability of evidence-based protocols for the management of CRS and neurological events will be critical to ensure an acceptable safety profile and treatment adherence. The accumulation of more data on the mechanisms and natural course of the AEs along with additional evidence confirming the reliability of current AE management strategies may aid the development of outpatient dosing protocols.

Response rates for topotecan or lurbinectedin monotherapy as second-line treatments for chemotherapy-refractory SCLC range between 10% and 35%, with a median DOR of around 5 months for lurbinectidin [78, 79]. Updated results from the FIH trial of tarlatamab indicate a similar response rate but a notably favorable median DOR (12.3 months) [63], although final data from the phase 1 study are awaited to confirm these outcomes. The durability of these responses may translate into a survival shift, as suggested by the observed median OS data in the FIH study. A phase 2 study (DeLLphi-301) evaluating tarlatamab in patients with relapsed/refractory SCLC (NCT05060016) after two or more prior lines of treatment is expected to yield primary results in 2023, while the phase 3 DeLLphi-304 study, which aims to compare the efficacy of tarlatamab against SOC chemotherapy in patients with relapsed/refractory SCLC following platinum-based first-line chemotherapy, will shortly begin recruiting patients (NCT05740566).

Additional clinical data from the ongoing anti-DLL3 TCE studies will be necessary to further define the clinical safety and efficacy profiles of these agents. Studies of other TCEs suggest that efficacy can be impacted by naturally arising biological phenomena, such as downregulation of target antigen expression [80] and an increase in the frequency of regulatory T cells [81]. Although these phenomena have not been documented with tarlatamab so far, monitoring of patients through the longitudinal analysis of immune cells and tumor DLL3 expression could provide important insights. Another potential concern with many biologics including TCEs is the development of antidrug antibodies (ADAs), which can potentially neutralize therapeutic efficacy, alter drug pharmacokinetics, and cause drug-related toxicities [82]. In the FIH trial of tarlatamab, 10 of 97 (10.3%) evaluable patients developed anti-tarlatamab antibodies on therapy; two patients (2.0%) had pre-existing ADAs at baseline [63]. There was no apparent impact of ADAs on tarlatamab exposure or on the safety profile in these patients.

Tarlatamab is also being evaluated in first-line SCLC. An ongoing phase 1b (DeLLphi-303) study (NCT05361395) aims to investigate the safety and efficacy of quadruplet therapy with tarlatamab in combination with CE and a PD-L1 inhibitor (atezolizumab or durvalumab), followed by maintenance treatment with tarlatamab and a PD-L1 inhibitor in patients with ES-SCLC in the first-line setting. Another study arm in this trial will investigate the safety and efficacy of tarlatamab when used in combination with a PD-L1 inhibitor as maintenance-only treatment following SOC chemotherapy. Additionally, a phase 3 study that will evaluate tarlatamab as first-line treatment in ES-SCLC is scheduled to commence in 2024.

The therapeutic potential of tarlatamab could be further enhanced by transformation of the current inpatient-only administration protocol to an outpatient setting starting from the early treatment cycles to improve patient adherence and convenience. In the FIH (DeLLphi-300) study, tarlatamab-associated CRS could be managed with multiple strategies, as described previously. As we continue to gain a better understanding of the timing, development and management of AEs, and strategies for the rational employment of biomarkers, the early identification of patients who are most likely to develop serious AEs that can result from these approaches may eventually help in the realization of outpatient dosing.

### ADCs

ADCs were designed to be the “magic bullets” that would achieve targeted delivery of a toxic payload to tumor cells only, thereby avoiding systemic toxicities associated with conventional chemotherapy regimens. Additionally, the wide therapeutic scope of ADCs, given their ability to exert antitumor activity independent of a patient’s immune status, was considered a clear advantage. Unfortunately, the toxicities associated with the PBD warhead of Rova-T precluded repetitive dosing of this agent in many patients and are likely to have contributed ultimately to their failure to demonstrate superior efficacy over SOC options in later-phase clinical trials. It is also possible that the patient population selected for the Rova-T FIH phase 1 study was not as representative of real-world patient populations as those included in later studies. It will be of interest to see if other non–DLL3-targeting ADCs with distinct cytotoxic warheads that are currently being evaluated for the treatment of SCLC (NCT04152499 and NCT04826341) will be able to succeed where Rova-T did not.

The next generation of ADCs is being designed to address some of the limitations observed with earlier generations. Enhanced antibody formats with new linkage technologies, improved stability profiles, and an optimized drug-antibody ratio aim to improve pharmacokinetics and expand the therapeutic window [83–85]. Similarly, structural improvements that “miniaturize” antibodies by the removal of the Fc segment, peptide-drug conjugates, and recombinant antibody fragments whose smaller size can potentially facilitate tumor penetration or uptake by tumor cells have the potential to make ADCs an attractive option for solid tumor therapy [86, 87]. ADCs may be well-suited for inclusion in rational combinatorial approaches with immunotherapy in solid tumors. ADC payloads released from dying tumor cells can directly prime dendritic cells and recruit effector cells to the vicinity of the tumor [83], suggesting that ADCs and immunotherapy combinations can potentially synergize to improve antitumor efficacy [85].

## Challenges and future perspectives

### Predictive/prognostic value of DLL3 and other biomarkers

Rova-T, tarlatamab, and other agents have shown specificity for DLL3-expressing cells and tumors in preclinical studies. It is reasonable to assume that the preselection of patients with high DLL3 expression would improve the efficacy of DLL3-targeting therapies. Indeed, in the FIH study of Rova-T, an exploratory subanalysis revealed that patients with high tumoral expression of DLL3 (≥ 50% of DLL3-expressing tumor cells) had improved ORR (35% vs 0%) and disease control (90% vs 60%) compared with DLL3-low patients [31]. However, subsequent clinical studies of Rova-T, including those that recruited only DLL3-high patients and those that analyzed subsets of patients with high DLL3 expression, did not confirm DLL3 expression as a biomarker predictive of therapeutic response [6, 33]. Several explanations have been put forward to explain this discrepancy, including the limited patient numbers in the FIH study and differences in the IHC techniques used to measure DLL3 expression [6]. Another theory proposes that the lack of response in DLL3-high patients may be related to suboptimal drug concentrations at the tumor site as a result of degradation of Rova-T in the peripheral circulation [88]. Additionally, the use of tumor biopsy IHC as a technique to assess tumoral DLL3 expression has certain drawbacks, including the lack of contemporaneous tumor biopsies—a potential issue in a rapidly progressive carcinoma, such as SCLC. The variability in DLL3 expression between the primary tumor and metastases could also confound interpretation [89].

The predictive role of DLL3 expression levels in determining response to DLL3-targeting therapies continues to be a matter of investigation. Exploratory analyses in the ongoing trials of tarlatamab in SCLC may shed additional light on this. Newer molecular techniques, such as the analysis of circulating tumor cells (CTCs) and/or circulating tumor nucleic acids, may allow for a real-time, noninvasive sequential analysis of DLL3 [90, 91]. Recent developments, such as the identification of DLL3 + /CD45– CTCs as a dynamic marker potentially associated with response to treatment, represent another potential avenue for future research [92]. In a study of 48 patients with advanced SCLC, the detection of DLL3-expressing CTCs in the peripheral blood was associated with significantly poorer survival outcomes [90]. Other noninvasive methods of assessing DLL3 expression, such as immuno-positron emission tomography (immunoPET), have the ability to measure low levels of DLL3 expression in primary tumor sites as well as in sites of distant metastases in real time [89]. ImmunoPET techniques may ultimately improve patient selection and could provide early information on the efficacy of DLL3-targeting therapies. It is notable that clinical studies have so far provided conflicting results on the stability of DLL3 expression over time and over lines of therapy [27, 28]. This is a critical issue that needs to be resolved, as this may help inform the design of the most optimal strategies for the use of DLL3 as a biomarker and treatment target.

Recent technological advances, such as the mapping and quantitation of tumor-specific methylation patterns in circulating cell-free DNA in patients with SCLC, could potentially predict disease progression, facilitate patient stratification in clinical trials, and help optimize treatment strategies to maximize clinical benefit [93].

### SCLC subtypes

It has been proposed that SCLC be classified into four subtypes based on the expression levels of the transcription factors ASCL1, NeuroD1, YAP1, and POU2F3 as SCLC-A, SCLC-N, SCLC-Y, and SCLC-P, respectively [94]. In subsequent IHC studies, YAP1 protein expression was shown to be low across subtypes, thereby prompting a modified classification approach comprising the SCLC-A, SCLC-N, SCLC-P, and SCLC-I subtypes, with SCLC-I referring to an inflamed state with low expression levels of the other three transcription factors but high expression levels of genes related to human leukocyte antigen expression, IFN-γ activation, and immune checkpoint molecule expression [14, 95]. The clinical relevance of these subtypes is suggested by a subgroup analyses of data from patients receiving platinum-etoposide with atezolizumab in the IMpower133 trial. A trend toward improved OS was observed in patients with the SCLC-I subtype relative to that seen in patients with the other subtypes (18 months vs 10 months) [14]. It will be of interest to see whether DLL3-targeting therapies achieve better outcomes in patients with SCLC-A and SCLC-N, as these subtypes express higher levels of DLL3 [14, 96]. In addition, it can also be expected that SCLC subtypes will play an important role in the choice of the PDX model to be used in preclinical studies, as a patient selection criterion in clinical trials, and potentially, as an additional predictive marker [97].

### Combination strategies with other therapeutic approaches

Combining TCEs and ADCs with other therapies could potentially provide a multipronged approach to reduce drug resistance, improve treatment efficacy, and allow for the use of lower treatment doses, thereby improving the therapeutic index. Checkpoint inhibitors have been explored in combination with both ADCs and TCEs. In a preclinical murine SCLC tumor model, Rova-T in combination with anti–programmed cell death protein-1 (PD-1) enhanced antitumor activity even at subefficacious doses [98]. This strategy also enhanced the tumor expression of PD-L1 and MHC 1 and increased the proliferative potential of and granzyme B production by CD8 + T cells [98]. A phase 1/2 study that evaluated Rova-T in combination with the ICIs nivolumab and ipilimumab in heavily pretreated patients with ES-SCLC reported an ORR of 30% [34], which compares favorably to that reported with Rova-T (ORR: 18%) [31] or nivolumab monotherapy (ORR: 11.6%) [99]. However, combinatorial toxicity was a concern, as 50% of patients (3 of 6 patients) in the high-dose cohort experienced dose-limiting toxicities and more than 90% experienced grade ≥ 3 TEAEs [34]. While it was not possible to fully distinguish the TEAEs caused by Rova-T from those caused by nivolumab or ipilimumab, the most frequently occurring AEs such as thrombocytopenia, serosal effusions, fatigue, and anemia were also observed in the Rova-T monotherapy studies, suggesting that Rova-T likely contributed to the development of these AEs.

Combining checkpoint inhibitors with TCEs has the potential to mutually increase each agent’s antitumor efficacy. TCEs can induce upregulation of PD-1 and PD-L1 expression on immune and tumor cells, and the addition of PD-1 and PD-L1 inhibitors was associated with enhanced activity of both T cells and TCEs in hematological malignancies and solid tumors [100]. In preclinical studies, tarlatamab upregulated PD-L1 expression on SCLC tumor cells and enhanced the T-cell–mediated lysis of tumor cells when combined with an anti–PD-1 antibody [101, 102]. In addition to the quadruplet combination mentioned previously, a phase 1b study exploring the safety and tolerability of a combination of tarlatamab with an anti–PD-1 antibody for patients with progressive or recurrent SCLC is ongoing (NCT04885998).

Combining conventional chemotherapy with targeted immunotherapy is another approach that offers multiple synergies that can potentially amplify the antitumor effect of TCEs. Several classes of chemotherapy drugs, such as alkylating agents, taxanes, platinum-based agents, and nucleoside analogs, have the ability to potentiate the activity of immunotherapy by sensitizing tumor cells to the granzyme B produced by cytotoxic T lymphocytes upon engagement, by enhancing tumor antigen recognition, by inhibiting immune suppressive cells, by reducing tumor burden levels, and by inducing the rapid rebound proliferation of CD8 + T cells after chemotherapy [103, 104]. Despite these postulated synergies, Rova-T in combination with CE did not improve efficacy rates in frontline ES-SCLC in a small group of 26 patients [105]. While lower doses of the Rova-T–CE combination were found to be tolerable, the combination did not improve clinical outcomes (median OS, PFS, or ORR) compared with the response rates typically seen with CE alone [105]. A study to evaluate tarlatamab in combination with carboplatin, etoposide, and a PD-L1 inhibitor as first-line treatment in patients with ES-SCLC has been initiated (NCT05361395) and may yield additional insights on the utility of this approach.

### DLL3-targeting therapies in other NETs

DLL3 is a TAA of interest in other high-grade NETs. High levels of DLL3 expression have been observed in NECs of the cervix (81% of tumor samples), poorly differentiated gastroenteropancreatic cancer (76.9%), castration-resistant NEPC (76.6%), LCNEC (74.0%), and neuroendocrine bladder cancer (68.0%) [29, 106–108]. A phase 1/2 study that evaluated Rova-T in 200 patients with DLL3-expressing advanced tumors, including NECs and other NETs, revealed a consolidated ORR of 13% in patients with NEC/NET [109]. Tarlatamab is currently under evaluation for the treatment of NEPC (NCT04702737). In addition to patients with SCLC, the phase 1 study of HPN328 has enrolled patients with NEPC and other neuroendocrine neoplasms with some preliminary evidence of antitumor activity in these tumor types [72]. The phase 1 trial of BI 764532 is recruiting patients with DLL3-positive LCNEC and NEC of any origin in addition to patients with DLL3-positive SCLC [110].

## Concluding perspectives

DLL3 continues to be a target of interest in SCLC and other NETs, as evidenced by the multiple DLL3-targeting molecules that are currently being clinically evaluated and a host of preclinical agents that are beyond the scope of this review. The successful clinical development of DLL3-targeting agents may benefit from a deeper understanding of SCLC biology and the willingness to revisit the lessons from other therapies that have failed in SCLC.

DLL3-directed TCEs have exhibited encouraging initial efficacy and safety profiles in early clinical studies of patients with relapsed/refractory SCLC. The identification of markers of response and toxicity, the characterization of DLL3 as a dynamic biomarker, and the refinement of AE management approaches are important areas of development for many of the DLL3-targeting therapies.

An ongoing challenge for DLL3-targeting ADCs in SCLC will be improvement of the therapeutic index through a combination of strategies, including optimization of the dosing schedule, treatment intervals, treatment duration, linker design, and warheads [111]. ADCs with improved toxicity profiles, improved drug-to-antibody ratios, an ability to engage with low levels of surface antigens in solid tumors, and carefully selected payloads are required for the treatment of solid tumors such as SCLC.

Despite the many challenges, the emerging data with DLL3-targeting agents offer renewed hope for patients with metastatic SCLC (Additional File 1).

## Supplementary Information

**Additional file 1. **Author Article Summary: Dr. Rudin summarizes the key points of the publication.

## Data Availability

Data sharing is not applicable to this article, as no datasets were generated or analyzed during the current study.

## Abbreviations

ADA: Antidrug antibody
ADC: Antibody-drug conjugate
AE: Adverse event
ASCL1: Achaete-scute homolog 1
B-ALL: B-cell precursoracute lymphoblastic leukemia
CAR: Chimeric antigen receptor
CD: Cluster of differentiation
CE: Cisplatin or carboplatin and etoposide
CI: Confidence interval
CRS: Cytokine release syndrome
CTC: Circulating tumor cell
DLL3: Delta-like ligand 3
DOR: Duration of response
ES-SCLC: Extensive-stage small cell lung cancer
Fab: Fragment antigen-binding
Fc: Fragment crystallizable
FDA: Food and Drug Administration
FIH: First-in-human
ICI: Immune checkpoint inhibitor
IFN-γ: Interferon gamma
IgG: Immunoglobulin G
IHC: Immunohistochemistry
ImmunoPET: Immuno-positron emission tomography
IL: Interleukin
LCNEC: Large cell neuroendocrine carcinoma
LS-SCLC: Limited-stage small cell lung cancer
MedDRA: Medical Dictionary for Regulatory Activities
MHC: Major histocompatibility complex
NEC: Neuroendocrine carcinoma
NEPC: Neuroendocrine prostate cancer
NET: Neuroendocrine tumor
NHP: Nonhuman primate
NK: Natural killer
NOD SCID: Nonobesediabetic/severe combined immunodeficiency
ORR: Objective response rate
OS: Overall survival
PBD: Pyrrolobenzodiazepine
PD-1: Programmed cell death protein-1
PD-L1: Programmed death-ligand 1
PDX: Patient-derived xenograft
PFS: Progression-free survival
Ph: Philadelphia chromosome
PR: Partial response
RECIST: Response evaluation criteria in solid tumors
Rova-T: Rovalpituzumab tesirine
scFv: Single-chain variable fragment
SCLC: Small cell lung cancer
SD: Stable disease
SOC: Standard-of-care
TAA: Tumor-associated antigen
TCE: T-cell engager
TCR: T-cell receptor
TEAE: Treatment-emergent adverse event
TNF-α: Tumor necrosis factor alpha
TRAE: Treatment-related adverse event
TriTAC: Tri-specific T cell-activating construct
TTP: Time to tumor progression
