# Supplementary material for: Emerging therapies targeting the delta-like ligand 3 (DLL3) in small cell lung cancer
Source: J Hematol Oncol. 2023 Jun 24;16:66. doi: 10.1186/s13045-023-01464-y (PMC10290806; doi:10.1186/s13045-023-01464-y)
Supplement: Supplementary file 1 — Additional file 1. Author Article Summary: Dr. Rudin summarizes the key points of the publication. [file 13045_2023_1464_MOESM1_ESM.pptx]

## Slide 1
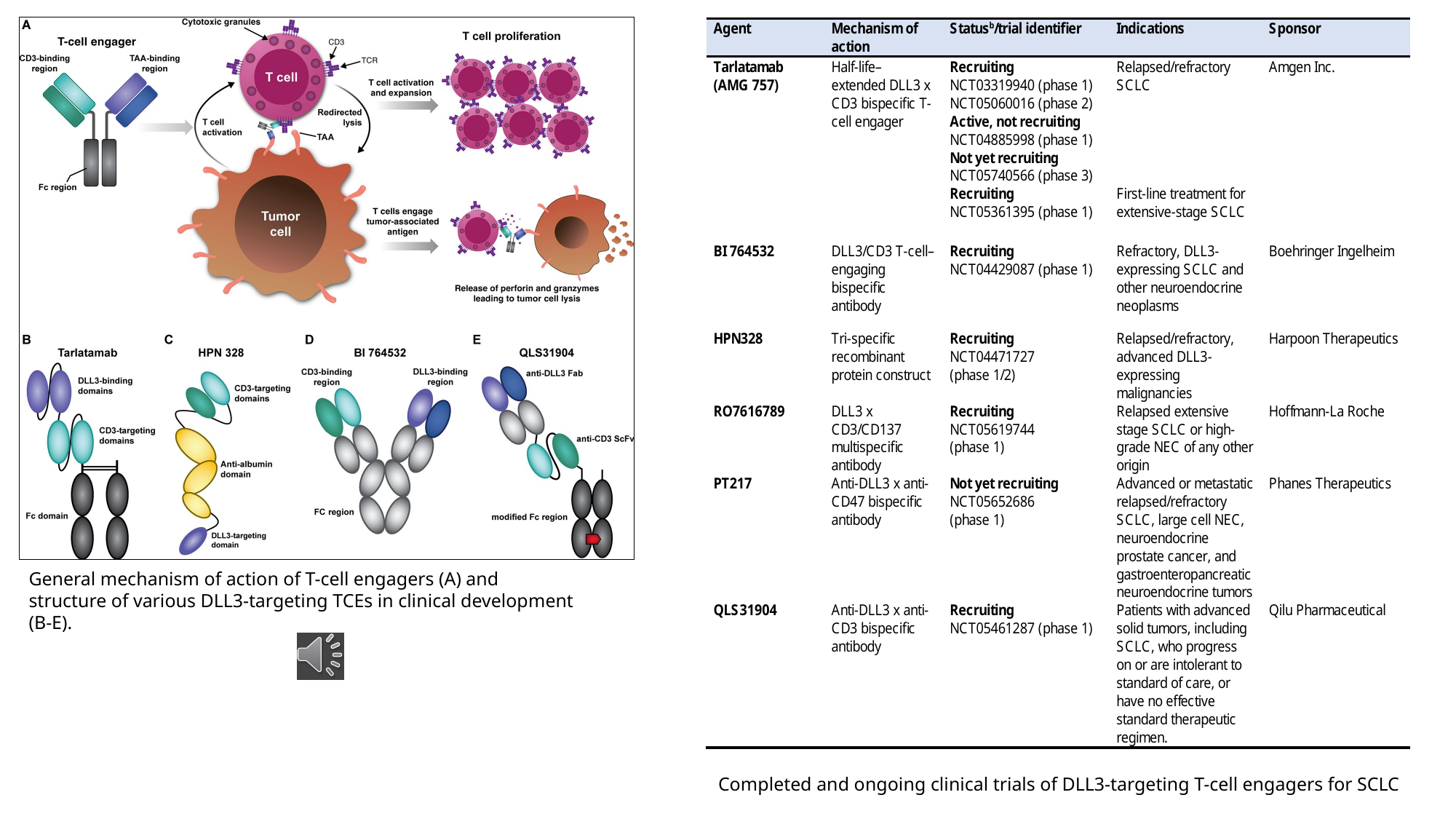

General mechanism of action of T-cell engagers (A) and structure of various DLL3-targeting TCEs in clinical development (B-E).
Completed and ongoing clinical trials of DLL3-targeting T-cell engagers for SCLC
